# Thermal Stability and Flammability Behavior of Poly(3-hydroxybutyrate) (PHB) Based Composites

**DOI:** 10.3390/ma12142239

**Published:** 2019-07-11

**Authors:** Henri Vahabi, Laurent Michely, Ghane Moradkhani, Vahideh Akbari, Marianne Cochez, Christelle Vagner, Estelle Renard, Mohammad Reza Saeb, Valérie Langlois

**Affiliations:** 1 Université de Lorraine, CentraleSupélec, LMOPS, F-57000 Metz, France; 2 Laboratoire Matériaux Optiques, Photoniques et Systèmes, CentraleSupélec, Université Paris-Saclay, 57070 Metz, France; 3 Université Paris Est, Institut de Chimie et des Matériaux Paris-Est, UMR 7182 CNRS-UPEC, 2, rue Henri Dunant, 94320 Thiais, France; 4 Aix Marseille University, CNRS, MADIREL UMR 7246, F-13397 Marseille, France

**Keywords:** poly(3-hydroxybutyrate) (PHB), flame retardancy, microcalorimetry of combustion

## Abstract

A series of samples based on poly(3-hydroxybutyrate) (PHB) containing five different additives were prepared and their thermal stability and flammability were discussed. The samples first underwent flammability screening by using Pyrolysis Combustion Flow Calorimeter (PCFC) analyses. Then, four samples were selected for further investigations. PHB composites containing sepiolite (Sep.) inorganic nanofiller, and also organic ammonium polyphosphate (APP) were examined for flammability and thermal behavior using PCFC, thermogravimetric analysis (TGA), flame test, and Differential Scanning Calorimetry (DSC) analyses. Moreover, burning behavior of samples were captured on a digital camera to give a deeper sense of their flammability character for comparison. The results revealed a significant improvement of flammability and thermal stability of composites, particularly in the presence of sepiolite with respect to the value obtained for unfilled PHB. Regarding TGA results, the char residue yield was increased to ca. 20.0 wt.% in the presence of sepiolite, while 0.0 wt.% was observed for PHB. PCFC measurements uncovered higher performance of PHB-Sep. sample as signaled by 40% reduction in the peak of heat release rate with respect to PHB. According to observations, PHB-Sep. sample showed non-dripping behavior with high capacity of charring in the presence of Sep. in a vertical flame test.

## 1. Introduction

Biodegradable biopolymer is a general term used for polymers that are synthesized from natural resources and can be degraded/decomposed by micro-organisms -what positioned them in front of fossil-based polymers in the frame of attention from an environmental perspective [1,2]. A recent survey revealed huge growth in the number of publications on bio-based polymers [3]. In polymer science and technology, two generations of biodegradable polyesters have been identified: poly(lactic acid) (PLA) and poly(hydroxyl alkanoate) (PHAs) [4]. Though PLA has already celebrated its maturity age, detailed analyses foresee a flourishing future of PHA in the global plastics market [4]. PHAs have exceptional characteristics such as optical activity, biocompatibility, a barrierity which is higher than PLA, and full biodegradability [5]. Moreover, synthesis of PHAs from bacteria eliminates the need for fermentation of food resources required in PLA production [6,7]. Furthermore, PHA is fully biodegradable in soil and marine water as well as in home compost. Researchers and engineers alike identified benefits of PHAs to keep it in competition with PLA thanks to the microbial features of PHAs [8]. On the other hand, high production cost, low thermal resistance, high flammability, poor mechanical properties compared to conventional polymers and the limited functionality of PHAs are the main reasons behind accelerative growth in research on PHAs [9,10,11]. Although the main application of PHAs is in the biomedical sector, a future prospect for PHAs can be imagined due to two of its exceptional characteristics: full biodegradability and a synthesis ability from bacteria [4]. In this sense, improvement of flammability properties of PHAs seems to be a priority for some applications [12]. Poly(3-hydroxybutyrate) (PHB) as the first member of PHAs family has been the candidate of many research programs [13]. Improvement of flame retardancy of some copolymers of PHB has been already studied by the incorporation of renewable raw materials, [14], melamine phosphate modified lignin [15], layered double hydroxides [16], kenaf fiber [17,18], halloysite nanotubes [19] aluminum phosphinate in combination with nanometric iron oxide and antimony oxide [10]. However, to the best of our knowledge, flame retardancy of PHB solely was not the subject of reports.

In this work, PHB and its various composites containing several additives were prepared and their behavior in terms of thermal decomposition and flammability was investigated. Following a preliminary work including twelve formulations, a series of composites containing sepiolite as well-known typical of mineral nanofillers, organic ammonium polyphosphate (APP) as phosphorus additive, and several natural additives including lignin and starch were developed individually and combinatorial. The amount of additive was kept constant while varying ingredient compositions in a PHB matrix to comparatively evaluate their flame scenario. It is well-known that sepiolite is a needle-like-shaped nanoclay and is biocompatible [20]. It has been already used in PHB and its copolymers in order to improve thermal and/or mechanical properties [21,22]. It was also proved that sepiolite as an inorganic biocompatible mineral can reveal promising features in combination with APP conventional flame retardant [23,24,25]. The combined use of the aforementioned additives from organic and inorganic families with micron and nano-size scales together with lignin and starch enabled us to understand the flammability behavior of PHB in different situations and provided a basis for understanding the degree to which PHB can be armed with additives to resist against fire. The prepared composites were subjected to a variety of characterizations. Pyrolysis combustion flow calorimeter (PCFC) was used as the first estimation technique to screen samples in terms of flammability. Moreover, thermogravimetric analysis (TGA), the direct flame test, scanning electron microscopy (SEM), size-exclusion chromatography (SEC), differential scanning calorimetry (DSC), and X-ray diffraction (XRD) measurements were performed.

## 2. Materials and Methods 

Poly(3-hydroxybutyrate) (PHB) powder was supplied by BIOMER (PHB T19, Krailling Germany). Ammonium polyphosphate (APP) was purchased from Clariant (Muttenz, Switzerland, Exolit AP 423, [NH_4_PO_3_]_n_, n > 1000, particle size ≈ 8 µm, specific surface area 1.1 m^2^/g). Sepiolite (Mg_4_Si_6_O_15_(OH)_2_.6H_2_O), namely Sep., was provided by Tolsa, Spain, (Pangel S9), and used without any modification. Starch (soluble, ACROS Organics™) was provided by Fisher Scientific (Hampton, NH, USA). Moreover, lignin (alkali) was purchased from Sigma-Aldrich (CAS Number: 8068-05-1).

PHB alone and together with additives was melt mixed in a Xplore conical twin screw microcompounder (MC 15 HT, Netherlands) at 175 °C and a rotor speed of 80 rpm during 5 min. First, PHB was fed into the mixing chamber and then after melting, additives were added. After the melt mixing was completed, the samples were casted as a thin film by using casting machine (Xplore- thickness: 50 µm, width: 3 cm). The sample names and compositions are given in Table 1. Loading percentage of additives was fixed at 15 wt.%. As mentioned earlier, sepiolite as a natural classic fire retardant in combination with APP and natural polymers was examined for flammability with PCFC first estimations. Lignin and starch were chosen as bio-based sources of carbon for flame retardant systems. These materials have already been used in PHB [26,27,28,29], however their combination with APP has never been reported.

The cross-section of selected samples was analyzed in a scanning electron microscope (SEM) manufactured by Carl Zeiss (Oberkochen, Germany) with a Field Emission Gun (FEG) at a magnification between 1.00K× et 5.00K× and at a working distance of 9–10 mm. The signal of the secondary electron was collected to scanning the sample. The voltage was 5 kV with a probe from 300 pA. The cross-sections were prepared by manual fracture in the transversal direction. Subsequently, the cross sections were metalized by a sputtering process with platinum (3.0 nm). The samples were also analyzed using an EDX (Energy-dispersive X-ray spectroscopy) microanalysis system (X-ray photon dispersion analysis). The EDX microanalysis system is an advanced Aztec EDS system, provided by Oxford Instruments (Abingdon-on-Thames, UK). The X detector is a 50 mm^2^ X-max SDD detector. Structural characterization was performed by X-ray diffraction (XRD) using a D8 advance Bruker diffractometer (Cu Kα radiation). Data were recorded over a 2θ range from 10 to 30° by step of 0.0102° at an incident wavelength λ of 1.54056 Å. From the patterns, the cell parameters a, b, and c of the orthorhombic unit cell were calculated from the maxima of the (040), (110), and (121) peaks according to Equation (1):

(1) $$\frac{1}{d^{2}}=\frac{h}{a^{2}}+\frac{k}{b^{2}}+\frac{l}{c^{2}}$$

where h, k, and l are the Miller crystallographic indexes and d is the interplanar spacing defined by Bragg’s law d = λ/(2sinθ ) with θ the scattering angle and λ the wavelength of the incident wave (=1.54056 Å). From these values, the lattice volume V was calculated according to the volume of an orthorhombic unit cell: V = a × b × c.

Thermal characterization was performed using Perkin Elmer Diamond Differential scanning calorimetry (DSC) with the following procedure: a first heating run from −60 to 200 °C with a heating rate of 20 °C/min was performed to determine the melting temperature (T_m_) and the melting enthalpy (ΔH_m_), followed by a cooling run to −60 °C with a cooling ramp of 200 °C/min. Then a second heating run from −60 to 200 °C at 20 °C/min was performed. The glass transition temperature (T_g_) was obtained in the second heating. The degree of crystallinity χ_c_ was calculated as a function of the real amount of PHB according to Equation (2):

(2) $$\chi c=\frac{1}{w_{PHB}}\times \frac{\Delta H_{m}}{\Delta H^{{}^{\circ}}{}_{m}}\times 100$$

where ΔH_m_ is the specific enthalpy of melting of the sample studied, w_PHB_ is the weight fraction of the PHB in the blend, and ΔH°_m_ represents the specific enthalpy of melting for the 100% crystalline PHB, taken as 146 J/g. 

Polymer molar masses were determined by Size-exclusion chromatography (SEC) using a Kontron 420 pump with 2 styragel columns connected in series which type is PL gel (mixte C) from polymer laboratories, and a Shodex RI-71 model refractive index detector (Japan). CHCl_3_ was used as eluent at a flow rate of 1.0 mL/min. A calibration curve was generated with polystyrene standards of low polydispersity purchased from Polysciences (Germany). Thermogravimetric analysis (TGA) was performed using a Setaram Labsys Evo thermogravimetric analyzer (France), under nitrogen with a heating rate of 10 °C/min.

Flammability properties were investigated using Pyrolysis Combustion Flow Calorimeter (PCFC) instrument (FTT Company, UK). The samples (1 to 4 mg) were heated at 1 °C/s from 20 °C to 750 °C in a pyrolyzer and the degradation products were conducted to another chamber and mixed with oxygen. Combustion took place at 900 °C. Each sample was tested 3 times and the related accuracy was around 5%. Vertical burning test (unnormalized) was also carried out on prepared film samples. Samples dimension was 120 mm × 25 mm × 50 µm. Samples were placed vertically inside a frame and exposed to a Bunsen burner flame for 3 s. A descriptive scheme of test is presented in Section 3.8. Moreover, digital video of tests was recorded and the selected images were also extracted. All videos are available in the Supplementary Materials.

## 3. Results

### 3.1. Premilinary Test by PCFC: Screening for Flammability Estimation

**Figure 1:**
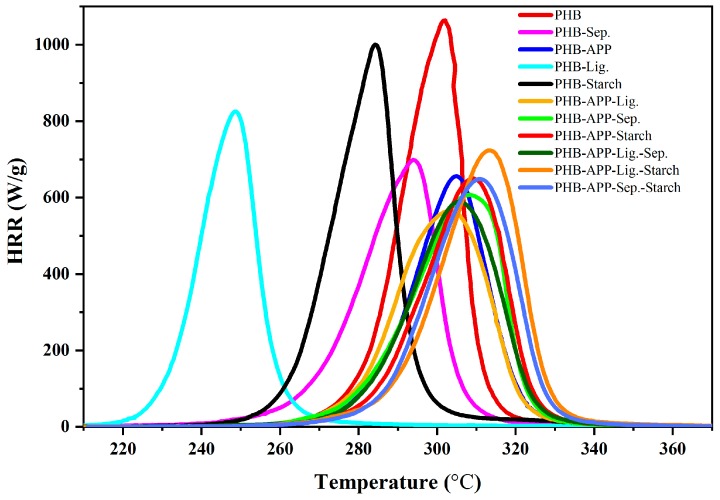
Heat Release Rate (HRR) curves obtained in PCFC tests.

A series of formulations containing APP, sepiolite, starch, lignin, and their combinations were prepared, Table 1. The goal of this preliminary work was to screening various formulations in terms of flammability and then selection of promising samples for the second-step investigation. Pyrolysis Combustion Flow Calorimeter (PCFC) is well known as a useful apparatus for screening the flammability of materials [30,31,32,33]. PHB and its composites defined in Table 1 were analyzed using PCFC. The obtained curves, heat of release rate (HRR) as a function of temperature, are presented in Figure 1. The presence of lignin and starch significantly decreased the temperature of peak of heat release (pHRR) rate and the reduction in pHRR was not significant in the presence of these additives in respect to that of PHB. The decrease in temperature of pHRR (T_pHRR_) can be attributed to degradation of PHB in presence of these fillers [28]. In the case of sepiolite, there is less difference between T_pHRR_ for PHB-Sep. and PHB. Moreover, the reduction of pHRR is more important. APP acts essentially in the condensed phase. The incorporation of APP was led to decrease in pHRR, close to the performance of sepiolite. However, it had no serious effect on T_pHRR_ compared to PHB. The combination of APP with sepiolite, lignin and starch increased T_pHRR_, while the value of pHRR was approximately near to that of other composites. Since the performance features of lignin and starch were less abundant than sepiolite, it was decided to conduct further investigation solely on PHB, PHB-Sep., PHB-APP, and PHB-APP-Sep. samples, Table 2.

### 3.2. Morphology Investigations (SEM)

**Figure 2:**
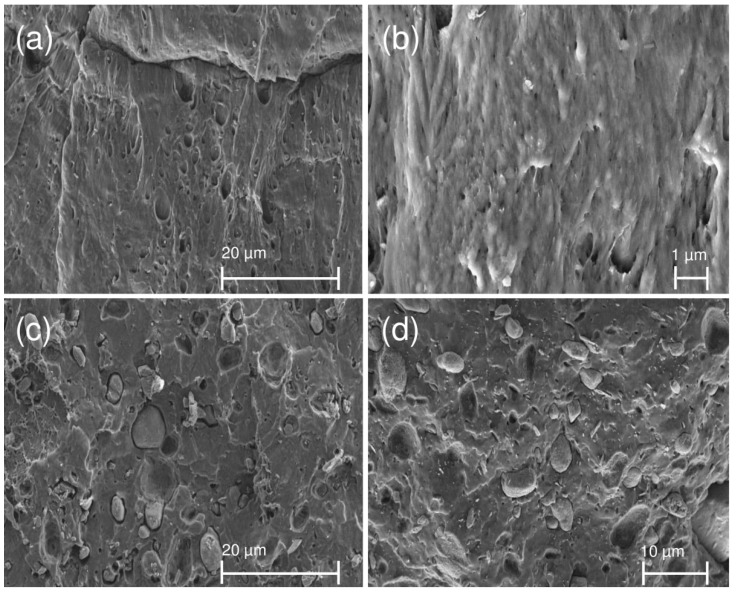
SEM images of PHB and its composites: (**a**) PHB; (**b**) PHB-Sep.; (**c**) PHB-APP; and (**d**) PHB-APP-Sep.

Figure 2 displays the SEM images of PHB and its composites. The micrograph of PHB shows a homogenous structure with only few structural defects, Figure 2a. Sepiolite nanoparticles were homogeneously dispersed in form of fibrous particle in PHB, Figure 2b. The size of APP particles was approximatively between 1 and 10 µm, Figure 2c. The adhesion of APP particles to the matrix seems not to be optimized. However, a better adhesion can be observed between APP and matrix in the case of a PHB-APP-Sep. sample, Figure 2d. Sepiolite particles are also homogeneously dispersed in nanometric size.

**Figure 3:**
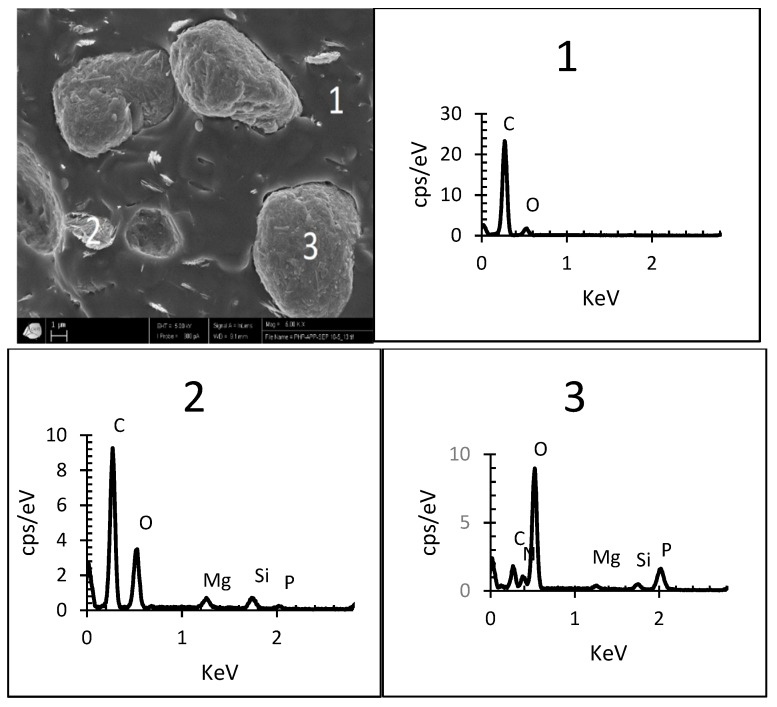
SEM images of PHB- APP-Sep. sample and analyzed zones by EDX.

EDX analyses performed on the PHB-APP-Sep. blend determined the elements characterizing sepiolite and APP (Figure 3). Based on SEM/EDX analysis in different areas, it is possible to better define the dispersion of sepiolite and APP (Table 3). In zone 1, the presence of peaks C and O confirms that the phase is mainly composed of PHB. Within this matrix, fine sepiolite needles are dispersed in zone 2 where the Si and Mg elements detected by EDX analysis show the presence of sepiolite. In zone 3, the presence of the elements P, Si and Mg attest the presence of both APP and sepiolite. There would therefore be an aggregation of the two types of charge with PHB with a majority of APP.

### 3.3. Differential Scanning Calorimetry (DSC)

**Figure 4:**
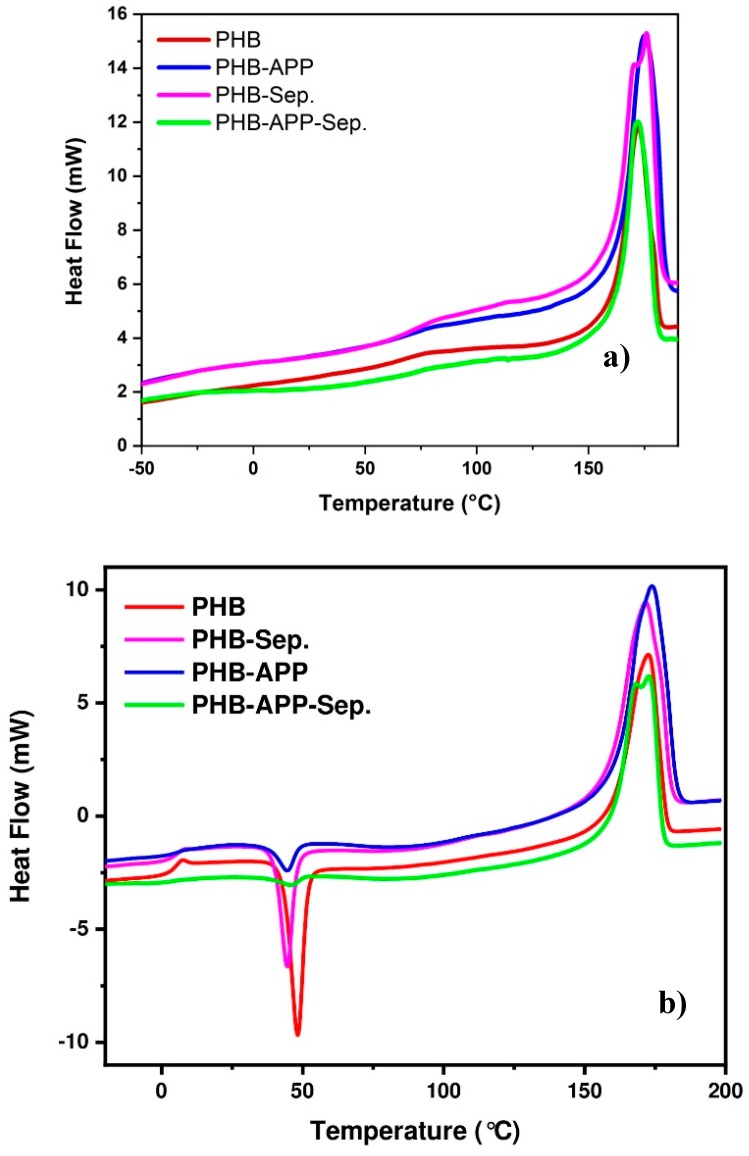
DSC curves for PHB, PHB-Sep., PHB-APP, PHB-APP-Sep. samples, (**a**) 1st heating (**b**) 2nd heating.

Differential scanning calorimetry (DSC) analysis was carried out on all samples and the obtained results recorded during the first and second heating cycles are presented in Figure 4 and Table 4. The glass transition temperature (Tg), the crystallization temperature (Tcc) and the melting temperature (T_m_) of PHB were found to be 5.3, 48.3 and 172.6 °C, respectively. These characteristic temperatures were not greatly modified by the incorporation of additives indicating that the presence of fillers does not affect the global morphology of PHB. However, the addition of additives, especially for sepiolite, led to a decrease in the crystallinity of PHB from 58 to 48 °C and probably led to a change in the size of the crystalline zones. The PHB-Sep blend display double melting peaks at 171.9 and 175 °C compared to pure PHB with one single melting peak at 172.2 °C. This is while T_m_ were affected more obviously due to sensitivity of PHB melting to additive addition. The presence of double endothermic melting peaks in the PHB composites was ascribed to the melt recrystallization mechanism [34].

All thermograms showed a crystallization peak between 45 °C and 48 °C during the second heating. This peak occurring after glass transition (T_g_) is due to a resumption of mobility of the molecules allowing the end of unfinished crystallization of the polymer during rapid cooling. In the presence of APP and sepiolite, the ΔH_Tcc_ of PHB decreased. When using both APP and sepiolite, this peak lost its amplitude (signaled by a decrease in ΔH_Tcc_), compared to the peak appearing for PHB. Such a behavior was completely changed by addition of APP to the formulation due to the pace formation of PHB crystals formed during the cooling process. All in all, the presence of additives did not affect Tg and Tm of PHB. Moreover, the decrease of crystallization enthalpy during the second heating rate was also inferred on account of the presence of additives and formation of crystalline domains during the cooling process after the first heating step.

### 3.4. X-Ray Diffraction

**Figure 5:**
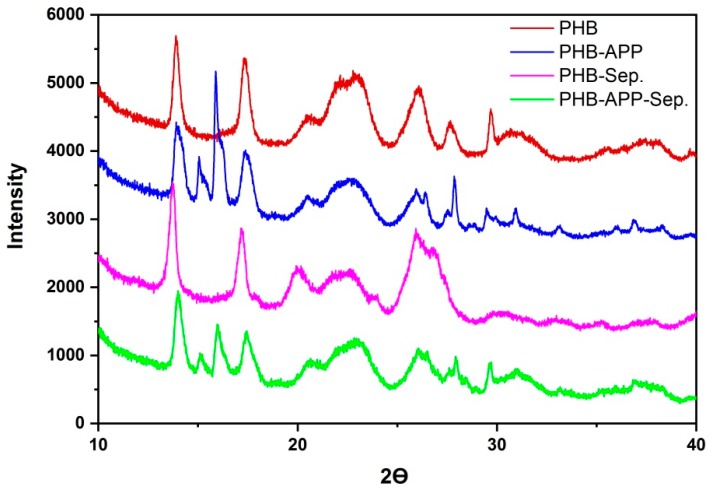
XRD patterns obtained from PHB; PHB-APP; PHB-Sep.; PHB-APP-Sep.

The crystal structure of PHB and the blends were analyzed by the Wide-angle X-ray scattering (WAXD) of α-form crystals, Figure 5. The two peaks, at about 15° and 16° for PHB-APP and PHB-APP-Sep. are diffraction patterns corresponding to APP rather than PHB crystals. It is obvious that the diffraction patterns and diffraction angles of the four specimens are almost similar (Table 5). The lattice parameters calculated by using interplanar spacing of planes (040), (110), and (121) in WAXD are shown in Table 6. The lattice parameters for PHB-Sep. are slightly different from those of PHB but the lattice for PHB-APP and PHB-APP-Sep. are similar to those of PHB. Particles of sepiolite slightly affected the basic crystal lattice. These results were supported by the lower values of melting enthalpy in the presence of sepiolite, which was determined by DSC.

### 3.5. Size-Exclusion Chromatography (SEC)

The impact of the incorporation of additives into the PHB was further studied by SEC. The obtained values in terms of number-average molecular weight (Mn), weight-average molecular weight (Mw), and polydispersity index (PDI) are given in Table 7. The M_n_ and M_w_ values were decreased in the presence of APP and sepiloite, either alone or combinatorial. However, the decrease in M_w_ was less important than M_n_ for all samples. Such a fall in Mn was more significant in the presence of sepiolite than APP. It was also possible that the blending procedure in the presence of additives contained small quantity of water that assisted in chain scission [6].

### 3.6. Thermogravimetric Analysis (TGA)

**Figure 6:**
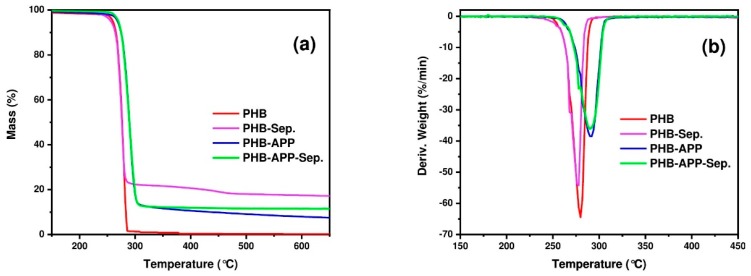
TGA thermograms of PHB and blends under nitrogen atmosphere. (**a**) TGA (**b**) DTG curves.

Figure 6 displays TGA (a) and derivative thermogravimetric (DTG) (b) thermograms of the PHB and its composites under nitrogen atmosphere. The important parameters extracted from these curves are presented in Table 8. First, it is apparent from the curves that PHB and its composite samples reveal a one-stage decomposition behavior under nitrogen atmosphere. The onset temperature of thermal decomposition (T_10%_, temperature at which 10% weight loss takes place) was 264 °C for the PHB. Another phenomenon was recognized to take place around 300 °C, where PHB was completely degraded and no residual mass was remained. The main mechanism of thermal decomposition of PHB corresponds to β-elimination of PHB chains that facilitates the formation of crotonic acid, dimeric, trimeric and tetrameric volatiles [35,36,37,38]. The presence of additives did not substantially decreased the T_onset_, in turn surprisingly led to a shift in decomposition temperature from 265 °C to 273 °C in the presence of APP and APP/Sep. Monitoring the maximum rate decomposition temperature from DTG curves (T_max_) unraveled that T_max_ shifted to higher temperatures in the presence of APP and APP/Sep. Starting from 310 °C, the thermal stability of PHB-Sep. sample was significantly higher than other samples. This trend was observed until the end of TGA measurements. The char residue at 800 °C was 16 wt.% for PHB-Sep. sample, against 6 wt.% and 11 wt.% for PHB-APP and PHB-APP-Sep. samples, respectively. It is well known that the presence of a flame retardant, especially a phosphorus one, can affect onset the temperature of a polymer [39]. Surprisingly, sepiolite and APP did not decrease the T_onset_ of PHB. Even by combination of APP and sepiloite, T_onset_ was shifted to a higher temperature.

### 3.7. Pyrolysis Combustion Flow Calorimeter (PCFC)

**Figure 7:**
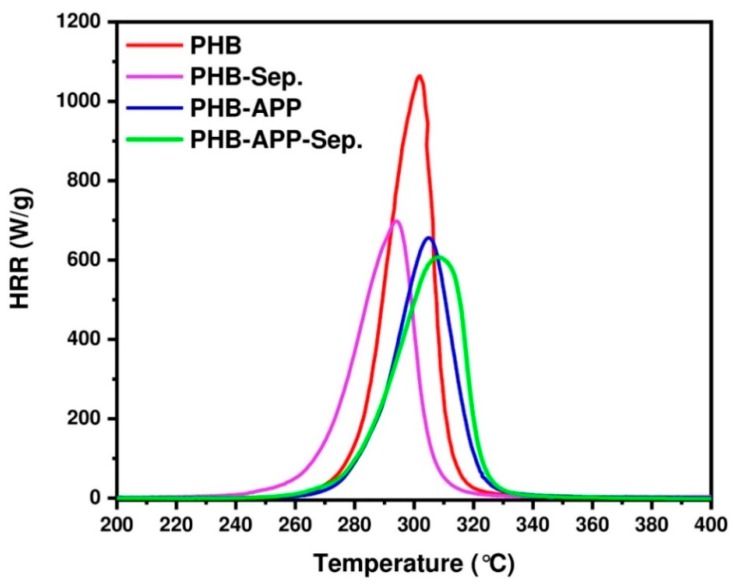
Heat Release Rate (HRR) curves obtained in Pyrolysis Combustion Flow Calorimeter PCFC tests.

The flammability of PHB was studied and then compared with those of composites containing APP, Sep. and APP-Sep. using PCFC test. Figure 7 shows HRR curves of the studied samples, and the important data are extracted from these curves and summarized in Table 9. The temperature of pHRR was slightly increased in the presence of APP as well as when combination of APP and Sep. was used. Changes observed in PCFC curve of PHB upon introduction of APP, Sep., and APP/Sep. is significant, implying the sensitivity of flame retardancy of PHB to additives used in this work. A closer look at data reveals that the peak of HRR (pHRR) significantly dropped from 1064 to 656 W/g by addition of Sep. to PHB, showing a reduction of 38%. In the presence of APP, however, the pHRR reached the value 699 W/g (34% reduction compared to value for PHB). The combination of sepiolite and APP promisingly led to a significant drop in pHRR by 43%. The value of THR (total heat release) slightly decreased for composite samples compared to PHB. The lowest THR value was recognized for PHB-Sep. sample, taking value of 17 kJ/g. The use of APP increased THR value due to the contribution of APP function into gas phase. The temperature assigned to pHRR point for samples was almost between 302 and 308 °C, expect for PHB-Sep for which the temperature was 291 °C, which makes a similar sense to TGA measurements. It should be noticed that even with a peak of HRR at 600 kW/m^2^, these samples can be considered as being flammable. However, the significant reduction in pHRR shows improvement in flammability behavior of these composites with respect to PHB. Although PCFC gives some indication about flammability of material, it is not a real fire test such as cone calorimeter. Therefore, it was decided to observe the real behavior of samples in front of direct flame using a set-up, which was adapted to the samples. 

### 3.8. Vertical Burning Test

**Figure 8:**
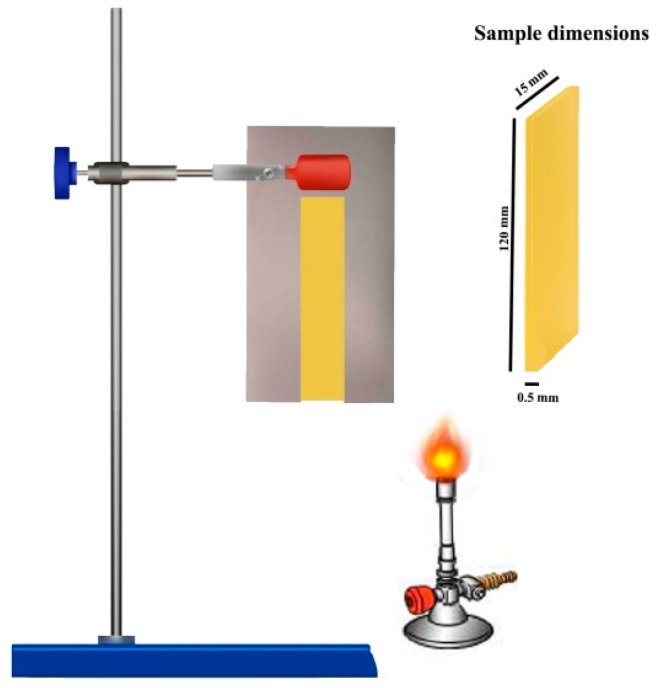
Illustration of flame test Set-up.

**Figure 9:**
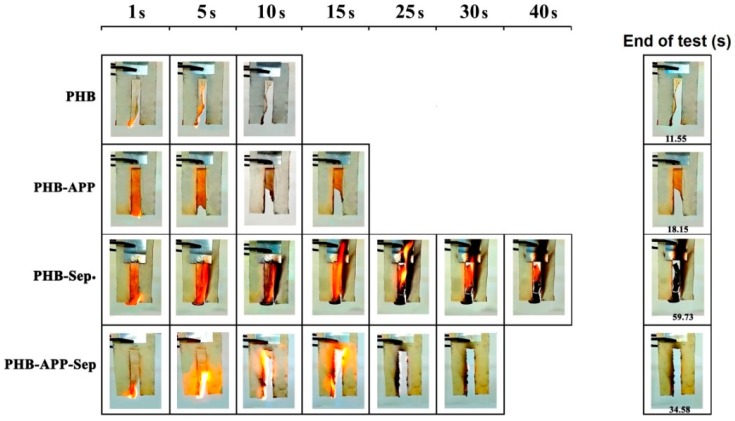
Evolution of flame propagation during vertical burning test for all samples.

A vertical burning test was performed according to the method described earlier. Figure 8 shows the set-up used, while Figure 9 displays the digital photos taken at different times of test (All videos are available in Supplementary Materials). PHB was completely burned in 10 s and the time to which flame got dormant was 11.5 s. Incorporation of APP increased the time of flame out to 18 s and featured the autoextinguible behavior observed. The PHB-APP sample revealed high dripping behavior. The combination of APP and Sep. led to increase in time of flameout to 35.5 s; however, this sample completely burned with dripping behavior and without remaining residue. Surprisingly, the time of flame out significantly increased for the PHB-Sep. sample, reaching 59.7 s. A high amount of residue was remained at the end of the test and dripping behavior was again observed for this sample during the test. Except PHB-Sep., all samples showed dripping behavior during the flame test (see videos in Supplementary Materials, Video S1–S4). Although vertical test has not been recognized yet as a normalized standard fire test, our observations revealed that the behavior of different samples can be compared in terms of the time takes to inflammation and dripping. Moreover, by using this test the quantity and the integrity of final char of the PHB/Sep. sample was properly understood.

## 4. Conclusions

The flammability and thermal stability behavior of PHB was investigated under the influence of the addition of organic ammonium polyphosphate (APP) as a conventional flame retardant, sepiolite as a inorganic biocompatible flame retardant, and lignin and starch. The preliminary assessment of flammability by PCFC confirmed that lignin and starch were the reason for high flammability of PHB. Then, investigations were continued by eliminating formulations in which lignin and starch were included. In the second phase, DSC results revealed that the presence of APP and sepiolite had no significant effect on glass transition and melting temperatures. TGA results showed that the presence of additives does not reduce the onset temperature of decomposition. In the case of combination of APP and sepiolite, the onset temperature was increased. The presence of sepiolite led to a significant increase in char residue, around 16 wt.% against 0 wt.% for pure PHB. PCFC results revealed the efficiency of sepiolite and its combination with APP to reduce peak of heat release rate, while vertical burning test clearly showed a huge difference behavior between PHB-Sep. and other samples. The peak of HRR of PHB was decreased from 1064 W/g to 656 W/g in presence of sepiolite (38% of reduction). The combination of sepiolite and APP was significantly affected the pHRR of PHB (42% of reduction).

## Figures and Tables

**Table 1:** Names and compositions of the PHB-based composite samples prepared in this study.

Number	Sample Code	PHB	Sepiolite	APP	Lignin	Starch
1	PHB	100	0	0	0	0
2	PHB-Sep.	85	15	0	0	0
3	PHB-APP	85	0	15	0	0
4	PHB-Lig.	85	0	0	15	0
5	PHB-Starch	85	0	0	0	15
6	PHB-APP-Lig.	85	0	10	5	0
7	PHB-APP-Sep.	85	5	10	0	0
8	PHB-APP-Starch	85	0	10	0	5
9	PHB-APP-Lig.-Sep.	85	2.5	10	2.5	0
10	PHB-APP-Lig.-Starch	85	0	10	2.5	2.5
11	PHB-APP-Sep.-Starch	85	2.5	10	0	2.5

**Table 2:** Names and compositions of selected samples after screening study.

Sample Code	PHB	Sepiolite	APP
PHB	100	0	0
PHB-APP	85	0	15
PHB-Sep.	85	15	0
PHB-APP-Sep.	85	5	10

**Table 3:** EDX analysis of PHB-APP-Sep. sample on different areas.

Area Number	Elements (%wt.) Normalized at 100					
	C	O	Si	Mg	P	N
Area 1	90.7	9.0	0.3	-	-	-
Area 2	66.4	24.9	4.6	2.5	1.6	-
Area 3	14.4	54.8	2.7	1.1	15.0	12.0

**Table 4:** DSC curves data of PHB and all composites (The values of enthalpy of melting for blends are normalized in compositions).

Sample	1st Heating			2nd Heating			
	T_m1_ (°C)	ΔH_Tm1_ (J/g)	Χc (%)	Tg (°C)	Tcc (°C)	ΔH_Tcc_ (J/g)	T_m2_ (°C)
PHB	172.2	85.4	58.2	5.30	48.3	36.7	172.6
PHB-APP	174.9	84.6	57.7	6.50	44.6	5.0	173.9
PHB-Sep.	171.9	70.8	48.3	5.70	44.6	14.2	171.5
PHB-APP-Sep.	175.9	73.3	50.0	4.15	45.9	2.4	172.9

**Table 5:** XRD peaks of PHB and blends.

Sample Code	2θ *(°)*					
	(020)	(110)	(021)	(111)	(121)	(040)
PHB	13.91	17.41	20.46	23.06	26.06	27.66
PHB-APP	14.02	17.41	20.41	22.94	25.98	27.63
PHB-Sep.	13.75	17.26	19.93	22.66	26.07	27.04
PHB-APP-Sep.	14.04	17.42	20.54	23.04	26.05	27.83

**Table 6:** Lattice constants and lattice volume of PHB and blends.

Sample Code	Lattice Constants (Å) & Lattice Volume (Å)			
	a	b	c	v
PHB	5.54	12.88	5.87	4.19
PHB-APP	5.54	12.90	5.92	4.23
PHB-Sep.	5.54	13.17	5.78	4.20
PHB-APP-Sep.	5.54	12.81	5.90	4.19

**Table 7:** Molecular weight of PHB and its corresponding blends.

Sample Code	M_n_ (g/mol)	M_w_ (g/mol)	PDI
PHB	106,000	254,400	2.4
PHB-APP	72,000	216,000	3.0
PHB-Sep.	55,000	148,500	2.7
PHB-APP-Sep.	69,000	186,300	2.7

**Table 8:** TGA parameters PHB and blends in nitrogen (*maximum weight loss temperature, obtained from DTG).

Sample Code	T_10%_ (°C)	T_max_* (°C)	Residue (wt.%)
PHB	265	280	0
PHB-Sep.	264	278	16
PHB-APP	274	292	6
PHB-APP-Sep.	273	290	11

**Table 9:** Summary results of Pyrolysis Combustion Flow Calorimeter (PCFC) tests.

Sample Code	THR (kJ/g)	T_pHRR_ (°C)	pHRR (W/g)	Reduction in pHRR (%)
PHB	22	302	1064	-
PHB-Sep.	17	291	656	38
PHB-APP	20	305	699	34
PHB-APP-Sep.	18	308	607	42

## Supplementary Materials

The following are available online at https://www.mdpi.com/1996-1944/12/14/2239/s1, Video S1: flame test-PHB; Video S2: flame test-PHB-APP; Video S3: flame test-PHB-APP-Sep.; Video S4: flame test-PHB-Sep.
